# Association between the A46G polymorphism (rs1042713) in the β2-adrenergic receptor gene and essential hypertension susceptibility in the Chinese population: A PRISMA-compliant meta-analysis

**DOI:** 10.1097/MD.0000000000023164

**Published:** 2020-11-13

**Authors:** Liyuan Yan, Haipeng Wang, Pengfei Liu, Minghan Wang, Jingjing Chen, Xin Zhao

**Affiliations:** Department of Cardiology, the First Affiliated Hospital of Soochow University, Suzhou, Jiangsu, China.

**Keywords:** β2-adrenergic receptor, Chinese population, hypertension, meta-analysis, polymorphism

## Abstract

**Background:**

Recently, many studies have been conducted to investigate the relationship between the A46G polymorphism in the β2-adrenergic receptor (ADRB2) gene and essential hypertension risk in the Chinese population. However, the results of previous studies were conflicting.

**Objectives:**

The present study aimed to investigate the association between the ADRB2 A46G polymorphism and the risk of essential hypertension in the Chinese population.

**Methods:**

We performed a systematic search of possible relevant studies on PubMed, Embase, Ovid, Web of Science, China National Knowledge Infrastructure, Wanfang, and China Biology Medicine disc databases up to January 3, 2020. Two authors independently extracted information from included articles and assessed the quality of each study by the use of the Newcastle–Ottawa Scale. According to the extent of interstudy heterogeneity, either a random-effect model or a fixed-effect model was used to calculate the combined odds ratio (OR) and 95% confidence interval (CI).

**Results:**

Finally, 16 studies containing 3390 cases and 2528 controls were included in our meta-analysis. Significant associations were found between the ADRB2 A46G polymorphism and essential hypertension risk in the Chinese population under four genetic models: allele genetic model (OR: 1.14, 95% CI: 1.06–1.23, *P* = .001, *P*_heterogeneity_ = .09), homozygote genetic model (OR: 1.29, 95% CI: 1.11–1.51, *P* = .001, P_heterogeneity_ = .25), dominant genetic model (OR: 1.17, 95% CI: 1.05–1.32, *P* = .005, P_heterogeneity_ = .04), and recessive genetic model (OR: 1.21, 95% CI: 1.05–1.38, *P* = .007, *P*_heterogeneity_ = .72).

**Conclusion:**

The ADRB2 A46G polymorphism may increase the risk of essential hypertension in the Chinese population.

## Abstract 2

Supplemental Digital Content is available in the text

## 1 Introduction

Hypertension, 1 of the most prevalent chronic diseases globally, is a risk factor for most cardiovascular and cerebrovascular diseases.^[1–3]^ With the urbanization and economic growth of China, the hypertension prevalence in China is increasing.^[4]^ According to a nationally representative cross-sectional study published in 2018, 23.2% of Chinese adults aged ≥18 years had essential hypertension.^[5]^ Essential hypertension is considered to be a complex disorder resulting from the interaction of genetic and environmental factors.^[6]^ Although environmental factors, such as obesity, alcohol drinking, and sodium intake, act as risk factors for hypertension, the contribution of genetic factors to essential hypertension cannot be neglected.^[6,7]^ With the development of sequencing technology, more and more variants in specific genes have been found to be associated with essential hypertension.^[8,9]^

β2-adrenergic receptor (ADRB2), a G-protein-coupled receptor, is regarded to be involved in the regulation of blood pressure in humans.^[10]^ ADRB2 plays a potential role in blood pressure regulating by their action on vascular resistance, renin release, and renal sodium excretion.^[11–13]^ Several genetic polymorphisms have been identified in ADRB2, of which the A46G (rs1042713, Arg16Gly) variant is the most frequent.^[14]^ The Arg16→Gly substitution has been shown to enhance the agonist-mediated receptor downregulation.^[15]^ There have been many studies conducted to investigate the association between the ADRB2 A46G polymorphism and the risk of hypertension in the Chinese population.^[16–31]^ However, the results of previous studies were conflicting. Therefore, we performed the present systematic review and meta-analysis to further investigate the association between the ADRB2 A46G polymorphism and essential hypertension risk in the Chinese population.

## 2 Methods

### 2.1 Search strategy

We performed a systematic search to identify all relevant studies on PubMed, Embase, Ovid, Web of Science, Chinese National Knowledge Infrastructure, Wan Fang, and China Biology Medicine disc databases up to January 3, 2020. The following subject terms or suitable keywords were used to perform the search: ADRB2 gene, β2-AR, polymorphism, variant, mutation, and hypertension with languages limited to Chinese and English (see Supplemental Digital Content, which illustrates the search strategy on PubMed). In order to include all possible relevant articles, references of relevant review articles and case-control studies were also searched. Two reviewers (Liyuan Yan, MD, and Haipeng Wang, PhD) independently performed the search and downloaded the full text of any possible relevant studies. The ethical approval and consent from patients were not needed because our study was based on previous published case-control studies. The meta-analysis was performed according to the Preferred Reporting Items for Systematic Reviews and Meta-Analyses.

### 2.2 Study selection

Studies included in the present meta-analysis must meet the following criteria:

(1) studies investigated the relationship between the A46G polymorphism in the ADRB2 gene and essential hypertension;
(2) studies with an unrelated case-control design in the Chinese population;
(3) studies provided enough data for us to calculate ORs and 95% confidence interval (CIs);
(4) studies fit the Hardy-Weinberg equilibrium (HWE) test in controls.

When it comes to duplicate publications, we only included the article with larger sample size and more comprehensive outcome information than the others. Two reviewers (Liyuan Yan, MD, and Haipeng Wang, PhD) independently performed the selection of included articles, and any conflicts between 2 reviewers were judged by a third reviewer (Xin Zhao, PhD).

### 2.3 Data extraction

Two authors (Liyuan Yan, MD, and Haipeng Wang, PhD) independently conducted the extraction of useful data from all studies included in our meta-analysis. The following data were extracted from included articles: name of the first author, year of publication, ethnicities, geographic location, number of cases and controls, mean age of cases and controls, genotypes distribution, source of controls, genotyping methods, and results of the HWE test in controls. Any conflicts between 2 reviewers were discussed with a third reviewer (Xin Zhao, PhD).

### 2.4 Quality assessment

Two reviewers (Liyuan Yan, MD, and Haipeng Wang, PhD) independently evaluated the quality of articles by using the 9-point Newcastle–Ottawa Scale (NOS).^[32]^ Each study was evaluated based on 3 aspects, including selection, comparability, and exposure. If the total scores of a study were less than six, it would be excluded from our meta-analysis.

### 2.5 Statistical analyses

All statistical analyses were conducted using Stata version 15.0 (Stata Corporation, College Station, TX). We used the chi-square test to assess the HWE of genotype in controls of each included study. The *Q* statistic test was performed to assess the interstudy heterogeneity, and *P* < .10 was considered that there was significant interstudy heterogeneity. The *I*^2^ statistic was calculated to quantify the effect of statistical heterogeneity. The sensitivity test was also used to find the origin of interstudy heterogeneity. The combined OR and 95% CI were calculated to estimate the association between the ADRB2 A46G polymorphism and essential hypertension risk under either a fixed-effect model (Mantel–Haenszel method) or a random-effect model (DerSimonian-Laird method) according to the degree of the between-study heterogeneity. If *I*^2^ < 50%, a fixed-effect model was used. A random-effect model was used to combine the results of included studies when *I*^2^ ≥50%. The analyses were conducted under 5 genetic models: allele genetic model (G versus A), homozygote genetic model (GG versus AA), heterozygote genetic model (AG versus AA), recessive genetic model (GG versus AA+AG), and dominant genetic model (AG+GG versus AA). In order to test the reliability of the pooled results, the sensitivity analyses were performed. We used Egger test and drawn Begg funnel plots to assess the publication bias in our study. *P* ≥ .05 indicated that there was no significant publication bias in our study.

## 3 Results

### 3.1 Characteristics of included studies

**Figure 1:**
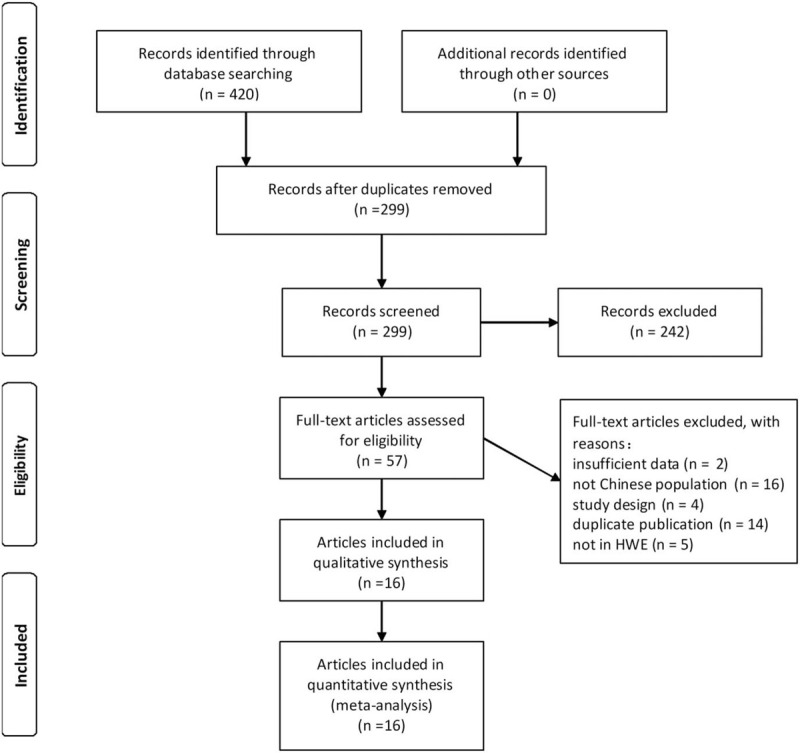
The PRISMA flow diagram of the study selection and exclusion.

The search of the 7 databases identified a total of 420 records. There were 299 records left after removing duplicate records. After screening the titles and abstracts of the 299 left records, 242 articles were excluded because they had nothing to do with the aim of our study. After reading the full text of the left 57 articles, 41 articles were excluded for the following reasons: 2 provided too limited data for us to calculate the pooled ORs and 95% CIs, 16 were not related to the Chinese population, 4 were not in an unrelated case-control design, 14 were duplicate publications, and 5 did not fit the HWE test in controls. Finally, a total of 16 articles (17 studies), containing 3489 cases and 2662 controls, were included in our meta-analysis. The complete selection and exclusion procedure of studies is shown in Figure 1. The characteristics of the included studies are shown in Table 1. Among the 17 studies, 12 studies were conducted in the Chinese Han population. The other 5 studies were conducted in the Chinese Yi, Hani, Kazak, Uyghur, and Tibetan minority populations, respectively. The NOS scores of included studies are shown in Table 2. All the 17 studies were of six or more NOS scores, which indicated the excellent quality of the included studies. The ADRB2 A46G polymorphism genotype distribution and allele frequency in cases and controls are shown in Table 3.

**Table 1:** Characteristics of the studies included for meta-analysis.

				Sample size		Age					
Author	Year	Ethnicity	Province	Case	Control	Case	Control	Genotyping method	Source of controls	NOS score	HWE test
Wu H^[16]^	2001	Han	Anhui	159	97	61.1 (9.2)	57.8 (10.5)	PCR-RFLP	HB	7	0.10
Zhu J^[17]^	2003	Han	Jiangsu	219	78	61.0 (14.2)	51.2 (17.9)	PCR-RFLP	HB	6	0.30
Wen CM^[18]^	2004	Han	Hubei	130	130	66.2 (9.7)	65.4 (10.8)	PCR-RFLP	HB	8	>0.99
Liang Y^[19]^	2004	Han	Liaoning	143	172	NA	NA	PCR-RFLP	PB	8	0.28
Chen BL^[20]^	2005	Han	Guizhou	131	40	66.3 (9.4)	64.4 (10.0)	PCR-RFLP	HB	8	0.07
Wu HY-Hani^[21]^	2006	Hani	Yunnan	172	133	52.2 (10.5)	50.6 (9.7)	Direct sequencing	PB	9	0.68
Wu HY-Yi^[21]^	2006	Yi	Yunnan	99	134	47.1 (10.4)	45.7 (7.5)	Direct sequencing	PB	9	0.94
Yu SF^[22]^	2008	Han	Henan	58	58	37.0 (6.5)^∗^		PCR-RFLP	PB	7	0.20
Liu BY^[23]^	2009	Han	Fujian	95	95	46.4 (5.9)	46.2 (5.7)	PCR-RFLP	PB	7	0.54
Wu HY^[24]^	2009	Han	Henan	96	196	50.6 (2.7)	42.5 (9.4)	PCR-RFLP	PB	8	0.07
ZhuM ^[25]^	2009	Han	Jiangsu	190	94	63.5 (4.6)	63.9 (4.8)	Gene chip	PB	9	0.40
Luo Q^[26]^	2010	Kazak	Xinjiang	347	217	48.5 (8.9)	44.9 (8.8)	PCR-RFLP	PB	8	0.44
Lou YQ^[27]^	2011	Han	Beijing	735	383	51.5 (9,5)	51.0 (7.7)	PCR-RFLP	PB	7	0.30
Gao JB^[28]^	2011	Han	Henan	102	116	58.4 (7.3)	NA	PCR-RFLP	HB	8	0.86
Zhang LP^[29]^	2012	Uyghur	Xinjiang	367	408	54.4 (10.3)	51.3 (10.2)	TaqMan	PB	8	0.33
Li XH^[30]^	2016	Tibetan	Gansu	332	257	47.8 (11.2)	46.4 (10.2)	SNaPshot mini-sequencing	PB	9	0.30
Yang R^[31]^	2016	Han	Jiangsu	114	54	NA	52.4 (16.1)	PCR-RFLP	HB	7	0.41

**Table 2:** The results of Newcastle-Ottawa Scale.

Study	Selection	Comparability	Exposure
Wu H (2001)^[16]^	^★★^	^★★^	^★★★^
Zhu J (2003)^[17]^	^★★^	^★^	^★★★^
Wen CM (2004)^[18]^	^★★★^	^★★^	^★★★^
Liang Y (2004)^[19]^	^★★★★^	^★^	^★★★^
Chen BL (2005)^[20]^	^★★★^	^★★^	^★★★^
Wu HY-Hani (2006)^[21]^	^★★★★^	^★★^	^★★★^
Wu HY-Yi (2006)^[21]^	^★★★★^	^★★^	^★★★^
Yu SF (2008)^[22]^	^★★★^	^★^	^★★★^
Liu BY (2009)^[23]^	^★★★^	^★★^	^★★^
Wu HY (2009)^[24]^	^★★★^	^★★^	^★★★^
Zhu M (2009)^[25]^	^★★★★^	^★★^	^★★★^
Luo Q (2010)^[26]^	^★★★★^	^★^	^★★★^
Lou YQ (2011)^[27]^	^★★^	^★★^	^★★★^
Gao JB (2011)^[28]^	^★★★^	^★★^	^★★★^
Zhang LP (2012)^[29]^	^★★★★^	^★^	^★★★^
Li XH (2016)^[30]^	^★★★★^	^★★^	^★★★^
Yang R (2016)^[31]^	^★★★^	^★^	^★★★^

**Table 3:** ADRB2 A46G polymorphism genotype distribution and allele frequency in cases and controls.

	Genotype (N)								Allele frequency (N)			
	Cases				Controls				Cases		Controls	
Study	AA	AG	GG	Total	AA	AG	GG	Total	A	G	A	G
Wu H^[16]^	74	53	32	159	44	37	16	97	201	117	125	69
Zhu J^[17]^	80	104	35	219	28	41	9	78	264	174	97	59
Wen CM^[18]^	36	68	26	130	53	60	17	130	140	120	166	94
Liang Y^[19]^	50	78	15	143	62	88	22	172	178	108	212	132
Chen BL^[20]^	12	87	32	131	4	25	11	40	111	151	33	47
Wu HY-Hani^[21]^	53	72	47	172	32	64	37	133	178	166	128	138
Wu HY-Yi^[21]^	53	34	12	99	28	66	40	134	140	58	122	146
Yu SF^[22]^	15	34	9	58	29	21	8	58	64	52	79	37
Liu BY^[23]^	26	40	29	95	34	48	13	95	92	98	116	74
Wu HY^[24]^	36	44	16	96	55	109	32	196	116	76	219	173
Zhu M^[25]^	65	97	28	190	32	49	13	94	227	153	113	75
Luo Q^[26]^	113	150	84	347	67	102	48	217	376	318	236	198
Lou YQ^[27]^	208	369	158	735	143	174	66	383	785	685	460	306
Gao JB^[28]^	28	53	21	102	47	53	16	116	109	95	147	85
Zhang LP^[29]^	100	196	71	367	130	209	69	408	396	338	469	347
Li XH^[30]^	98	180	54	332	80	134	43	257	376	288	294	220
Yang R^[31]^	40	46	28	114	21	23	10	54	126	102	65	43

### 3.2 Results of meta-analysis

**Figure 2:**
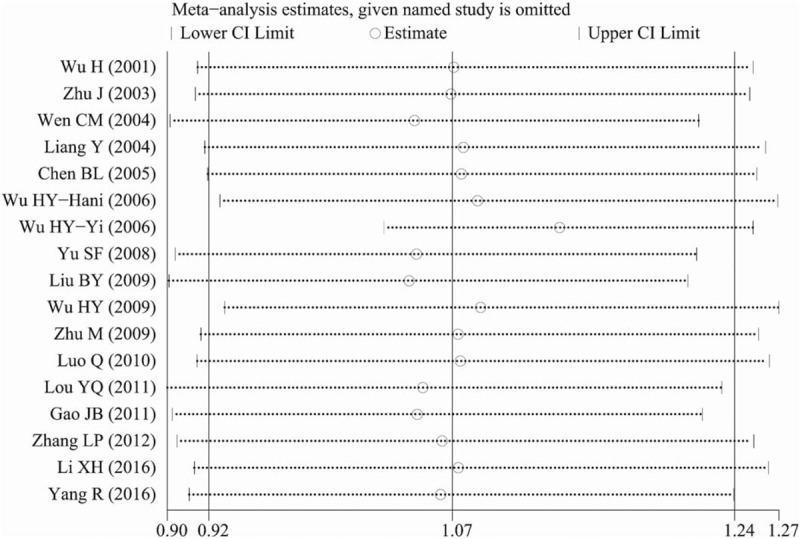
Sensitivity analysis of the pooled OR coefficients on the relationship between the ADRB2 A46G polymorphism and hypertension risk in 17 studies. ADRB2 = β2-adrenergic receptor, OR = odds ratio.

**Figure 3:**
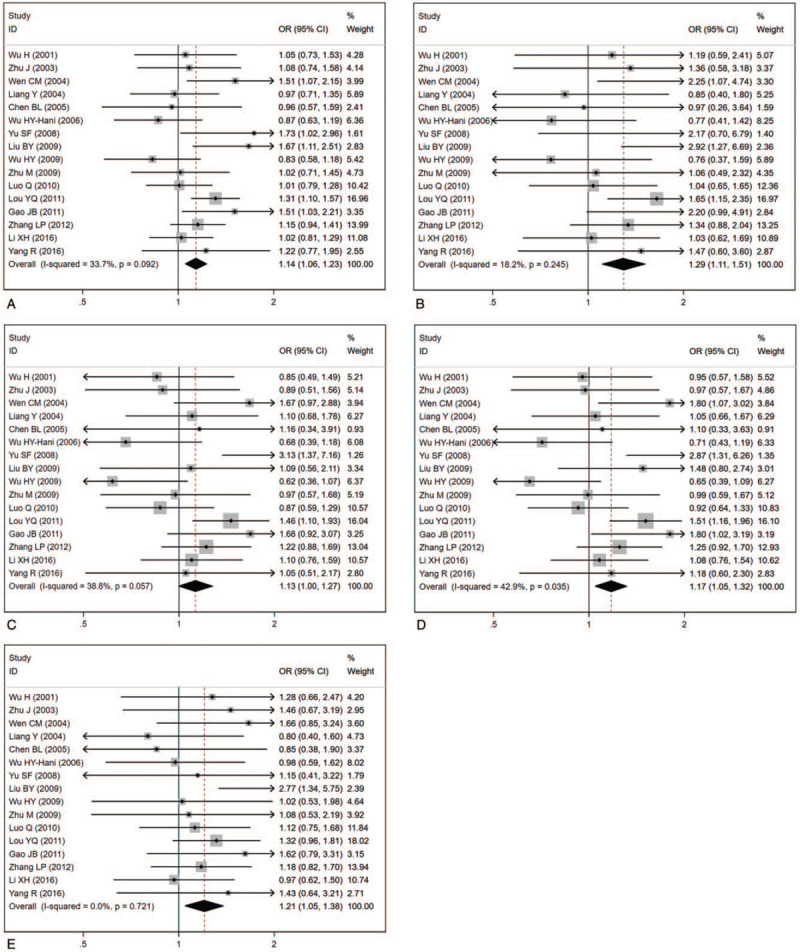
Forest plot from the meta-analysis on the association between the ADRB2 A46G polymorphism and hypertension risk. (A) allele genetic model; (B) homozygote genetic model; (C) heterozygote genetic model; (D) dominant genetic model; (E) recessive genetic model. ADRB2 = β2-adrenergic receptor, CI = confidence interval, OR = odds ratio.

Initially, we conducted a combination of the results of all 17 studies. There were no significant associations between the ADRB2 A46G polymorphism and hypertension risk under five genetic models: allele genetic model (OR: 1.07, 95% CI: 0.92–1.24, *P* = .38, *P*_heterogeneity_ < .001), homozygote genetic model (OR: 1.16, 95% CI: 0.88–1.52, *P* = .29, *P*_heterogeneity_ < .001), heterozygote genetic model (OR: 1.03, 95% CI: 0.83–1.26, *P* = .81, *P*_heterogeneity_ < .001), dominant genetic model (OR: 1.06, 95% CI: 0.85–1.33, *P* = .58, *P*_heterogeneity_ < .001), and recessive genetic model (OR: 1.14, 95% CI: 1.00–1.30, *P* = .052, *P*_heterogeneity_ = .09) (Table 4). There was great interstudy heterogeneity in the pooled analyses. Therefore, we conducted sensitivity analyses to discover the origin of the interstudy heterogeneity. As shown in Figure 2, the combined results greatly changed after omitting 1 study about the Chinese Yi minority population. Therefore, we performed pooled analyses without the study related to the Chinese Yi minority population. With this change, we found that there were significant associations between the ADRB2 A46G polymorphism and essential hypertension risk under four genetic models: allele genetic model (OR: 1.14, 95% CI: 1.06–1.23, *P* = .001, *P*_heterogeneity_ = .09), homozygote genetic model (OR: 1.29, 95% CI: 1.11–1.51, *P* = .001, *P*_heterogeneity_ = .25), dominant genetic model (OR: 1.17, 95% CI: 1.05–1.32, *P* = .005, *P*_heterogeneity_ = .04), and recessive genetic model (OR: 1.21, 95% CI: 1.05–1.38, *P* = .007, *P*_heterogeneity_ = .72) (Fig. 3A-3E and Table 4).

**Table 4:** Meta-analysis of the association between the ADRB2 A46G polymorphism and hypertension risk.

			Sample size		Test of association			Test of heterogeneity	
Genetic contrasts	Analysis	N	Case	Control	OR	95% CI	*P*	*P*_h_	*I*^2^ (%)
Allele model	Overall	17	3489	2662	1.07	(0.92, 1.24)	.38	<.001	72.0
	Without Wu HY-Yi	16	3390	2528	1.14	(1.06, 1.23)	.001	.09	33.7
	HB	6	855	515	1.23	(1.05, 1.45)	.01	.48	0.0
	PB	11	2634	2147	1.01	(0.83, 1.22)	.95	<.001	80.0
	Han	12	2172	1513	1.21	(1.10, 1.33)	<.001	.12	33.5
	Minority	5	1317	1149	0.84	(0.61, 1.16)	.29	<.001	86.8
Homozygote model	Overall	17	3489	2662	1.16	(0.88, 1.52)	.29	<.001	64.0
	Without Wu HY-Yi	16	3390	2528	1.29	(1.11, 1.51)	.001	.25	18.2
	HB	6	855	515	1.58	(1.12, 2.22)	.009	.73	0.0
	PB	11	2634	2147	1.02	(0.71, 1.46)	.92	<.001	73.9
	Han	12	2172	1513	1.47	(1.20, 1.81)	<.001	.35	10.3
	Minority	5	1317	1149	0.75	(0.42, 1.33)	.33	<.001	82.5
Heterozygote model	Overall	17	3489	2662	1.03	(0.83, 1.26)	.81	<.001	63.8
	Without Wu HY-Yi	16	3390	2528	1.13	(1.00, 1.27)	.05	.06	38.8
	HB	6	855	515	1.18	(0.91, 1.52)	.21	.39	4.3
	PB	11	2634	2147	0.97	(0.73, 1.27)	.80	<.001	73.9
	Han	12	2172	1513	1.20	(1.03, 1.40)	.02	.07	41.3
	Minority	5	1317	1149	0.78	(0.51, 1.19)	.25	.001	79.9
Dominant model	Overall	17	3489	2662	1.06	(0.85, 1.33)	.58	<.001	71.5
	Without Wu HY-Yi	16	3390	2528	1.17	(1.05, 1.32)	.005	.04	42.9
	HB	6	855	515	1.28	(1.00, 1.62)	.047	.37	7.8
	PB	11	2634	2147	0.98	(0.73, 1.32)	.91	<.001	79.6
	Han	12	2172	1513	1.27	(1.10, 1.47)	.001	.05	43.4
	Minority	5	1317	1149	0.76	(0.47, 1.22)	.26	<.001	85.7
Recessive model	Overall	17	3489	2662	1.14	(1.00, 1.30)	.052	.09	33.7
	Without Wu HY-Yi	16	3390	2528	1.21	(1.05, 1.38)	.007	.72	0.0
	HB	6	855	515	1.38	(1.02, 1.86)	.04	.85	0.0
	PB	11	2634	2147	1.06	(0.84, 1.33)	.64	.03	50.9
	Han	12	2172	1513	1.31	(1.09, 1.57)	.003	.63	0.0
	Minority	5	1317	1149	0.90	(0.64, 1.27)	.57	.03	63.0

We also performed subgroup analyses based on the source of controls and ethnicity. The detailed information was shown in Table 4. In subgroup analyses based on the source of controls, significant associations were observed between the ADRB2 A46G polymorphism and hypertension risk in the hospital-based group. Similar associations were also found between the ADRB2 A46G polymorphism and hypertension risk in the Han group, but not in the Chinese minority group.

### 3.3 Sensitivity analyses

**Figure 4:**
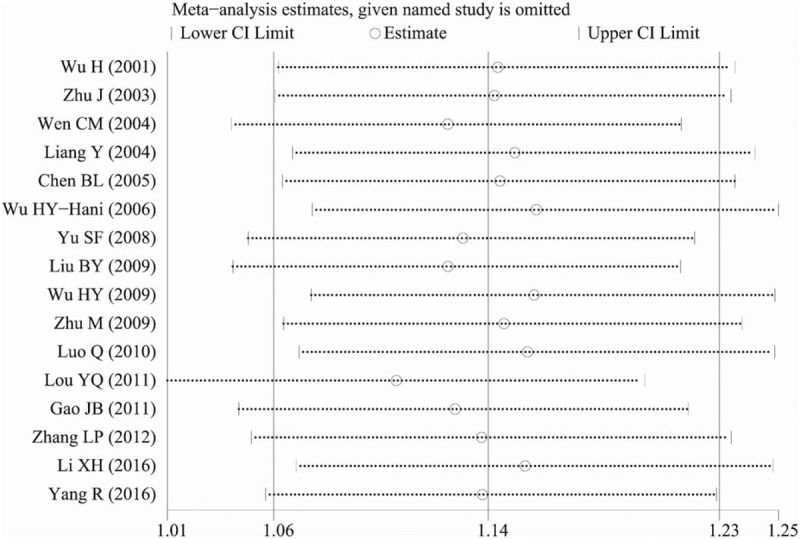
Sensitivity analysis of the pooled OR coefficients on the relationship between the ADRB2 A46G polymorphism and hypertension risk in 16 studies. ADRB2 = β2-adrenergic receptor, OR = odds ratio.

The sensitivity analysis was performed to explore whether the combined results would change quantitatively after omitting 1 study at each round of the analysis. As shown in Figure 4, there was no quantitative alternation of the combined results, which indicated the reliability of our results.

### 3.4 Publication bias

**Figure 5:**
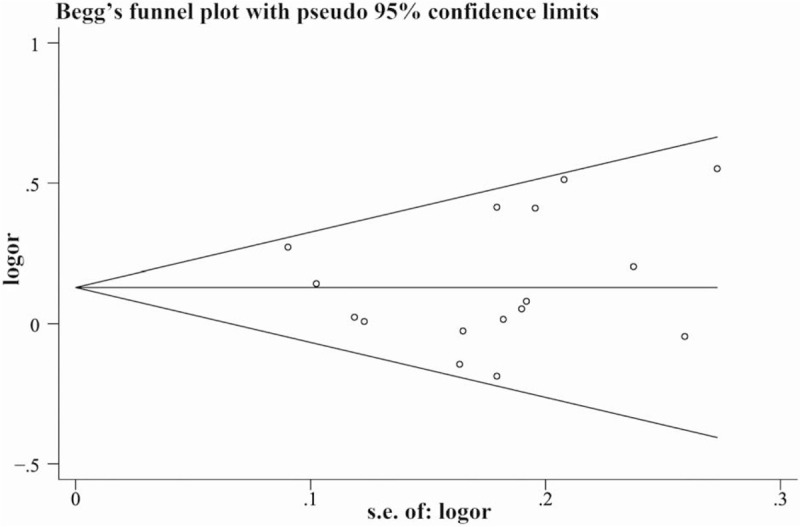
Begg's funnel plot with pseudo 95% confidence limits under the allele genetic model.

The publication bias is an inevitable problem when performing a meta-analysis. We used Egger test and drawn Begg funnel plots. As shown in Figure 5, the distribution of the 16 studies were symmetrically on the 2 sides, which indicated there was no publication bias in our present meta-analysis (Egger test: allele model: *P* = .89; homozygote model: *P* = .86; heterozygote model: *P* = .72; dominant model: *P* = .86; recessive model: *P* = .61).

## 4 Discussion

Essential hypertension is caused by the interaction of environmental and genetic factors.^[6]^ With the development of sequencing technology, more and more single nucleotide polymorphisms have been found to be related to hypertension susceptibility.^[9]^ The sympathetic nervous system contributes to the regulation of blood pressure by affecting cardiac output, the total peripheral vascular resistance, and sodium handling.^[33]^ The ADRB2 gene, as a pivotal adrenergic pathway gene of the sympathetic nervous system, plays an important role in cardiac output, air smooth muscle tone, and vasodilation.^[34]^ On the term of physiology, variants of the ADRB2 gene may result in hypertension by attenuating vasodilation, which leads to the increase of vascular resistance.^[33]^ The ADRB2 A46G polymorphism, which forms AA, AG, and GG 3 patterns, is 1 of the most studied single nucleotide polymorphisms in the ADRB2 gene.

So far, there have been many studies performed to investigate the relationship between the A46G polymorphism in the ADRB2 gene and the risk of essential hypertension in the Chinese population.^[16–31]^ However, the results Srevious studies were inconsistent. Several case-control studies suggested that the ADRB2 A46G polymorphism increased the risk of hypertension in the Chinese population. However, there was no significant association between the ADRB2 A46G polymorphism and hypertension risk in some other studies. There have been 2 meta-analyses published before exploring the relationship between the ADRB2 A46G polymorphism and essential hypertension susceptibility.^[35,36]^ The first 1 published in 2010 found that the ADRB2 A46G polymorphism was associated with hypertension risk in Mixed Africans. In this meta-analysis, only 2 studies in the Chinese population were included, and no significant association was found between the ADRB2 A46G polymorphism and hypertension risk in Asians.^[36]^ The other meta-analysis published in 2012, which contained 11 studies, indicated that the ADRB2 A46G polymorphism increased the risk of hypertension in the Han Chinese population under the homozygote and recessive model.^[35]^ There have been several studies published since 2012, and several studies conducted in the Chinese minority population were not included in previous meta-analyses.^[21,26,29–31]^ Therefore, we conducted the present meta-analysis to further investigate the association between the ADRB2 A46G polymorphism and the risk of essential hypertension in the Chinese population.

In pooled analyses of all 17 studies, there was no significant association between the ADRB2 A46G polymorphism and hypertension risk under five genetic models. However, we observed great heterogeneity among the included studies. One study^[21]^ related to the Yi minority population was found to result in great interstudy heterogeneity by using the sensitivity analysis. It may suggest that the ADRB2 A46G polymorphism plays a different role in hypertension risk in the Yi minority population, and more studies concerning the Yi minority are needed. After removing the above study, we conducted the meta-analysis of 5 ethnic groups (Han, Hani, Kazak, Uyghur, and Tibetan Chinese population), containing 3390 cases and 2528 controls. Moreover, we found that the ADRB2 A46G was correlated to the increased risk of essential hypertension risk under the allele, homozygous, dominant, and recessive genetic model. There was no significant interstudy heterogeneity under the homozygote genetic model and the recessive genetic model. No publication bias was observed in our study. Sensitivity analyses showed the reliability of our pooled results of 16 studies. In subgroup analyses, there were similar associations between the ADRB2 A46G polymorphism and hypertension risk in the hospital-based group and the Han group, but not in the population-based group or the Chinese minority group. We observed that all studies in the hospital-based group were conducted in the Han Chinese population. On the contrary, studies in the population-based group were related to different ethnic groups. The different genetic backgrounds of ethnic groups might result in great interstudy heterogeneity and negative combined results in the population-based group and the Chinese minority group.

Several limitations exist in our meta-analysis. First, we could not perform subgroup analyses of each ethnic group because there had not been enough eligible studies with regard to each minority. Second, there might be unpublished studies resulting in selection bias. Third, only studies wirtten in English or Chinese were included in our meta-analysis, which might result in selection bias. Fourth, the present meta-analysis did not investigate the gene-gene and gene-environment interactions on hypertension.

## 5 Conclusion

In conclusion, ADRB2 A46G polymorphism may increase the risk of essential hypertension in the Chinese population. More studies related to the Chinese ethnic minority population should be performed to help better reveal the association between the ADRB2 A46G polymorphism and hypertension risk in different Chinese ethnic groups.

## Author contributions

All authors contributed significantly to this study. Liyuan Yan and Haipeng Wang contributed equally to this work and should be considered as co-first authors. Xin Zhao, Liyuan Yan, and Haipeng Wang designed the study. Liyuan Yan and Haipeng Wang collected the data. Pengfei Liu analyzed the data. Minghan Wang and Jingjing Chen made the tables and figures. Xin Zhao, Liyuan Yan, and Haipeng Wang wrote the paper. All authors read and met the ICMJE criteria for authorship. All authors reviewed and approved the manuscript.

**Conceptualization:** Liyuan Yan, Haipeng Wang.

**Data curation:** Liyuan Yan.

**Formal analysis:** Liyuan Yan, Pengfei Liu.

**Methodology:** Liyuan Yan.

**Project administration:** Liyuan Yan.

**Resources:** Liyuan Yan, Xin Zhao.

**Software:** Liyuan Yan, Pengfei Liu, Minghan Wang.

**Supervision:** Haipeng Wang, Xin Zhao.

**Validation:** Haipeng Wang, Jingjing Chen, Xin Zhao.

**Visualization:** Pengfei Liu, Minghan Wang, Jingjing Chen.

**Writing – original draft:** Liyuan Yan.

**Writing – review & editing:** Haipeng Wang, Xin Zhao.

## Supplementary Material

Supplemental Digital Content
