# Supplementary material for: Association between the A46G polymorphism (rs1042713) in the β2-adrenergic receptor gene and essential hypertension susceptibility in the Chinese population: A PRISMA-compliant meta-analysis
Source: Medicine (Baltimore). 2020 Nov 13;99(46):e23164. doi: 10.1097/MD.0000000000023164 (PMC7668484; doi:10.1097/MD.0000000000023164)
Supplement: Supplemental Digital Content [file medi-99-e23164-s001.docx]

**Supplementary Materials: Association between the A46G polymorphism (rs1042713) in the β2-adrenergic receptor gene and essential hypertension susceptibility in the Chinese population: A PRISMA-compliant meta-analysis**

**Authors:** Liyuan Yan, MD^a1^; Haipeng Wang, PhD^a1^; Pengfei Liu, MD^a^; Minghan Wang, MD^a^; Jingjing Chen, MD^a^; Xin Zhao, PhD^a^*****

**Search strategy. Source: PubMed (Searched on: January 3, 2020).**

#1 β2-adrenergic receptor gene [All fields]

#2 β2-AR [All fields]

#3 hypertension [All fields]

#4 polymorphism [All fields]

#5 variant [All fields]

#6 mutation [All fields]

#7 #1 OR #2

#8 #4 OR #5 OR #6

#9 #3 AND #7 AND #8
